# The Action of Botulinum Toxin A on the Sternocleidomastoid Muscle: An Experimental Study on Rats

**DOI:** 10.1155/2022/2188783

**Published:** 2022-02-08

**Authors:** Themistoklis Vampertzis, Christina Barmpagianni, Chrysa Mpekiari, Rania Baka, Ioannis Zervos, Eleftherios Tsiridis, Nikiforos Galanis

**Affiliations:** ^1^School of Medicine, Aristotle University of Thessaloniki, Thessaloniki, Greece; ^2^Surrey and Borders Partnership NHS Trust, Leatherhead, UK; ^3^Laboratory of Anatomy and Histology, School of Veterinary Medicine, Faculty of Health Sciences, Aristotle University of Thessaloniki, Thessaloniki, Greece; ^4^Diagnostic Laboratory, School of Veterinary Medicine, Faculty of Health Sciences, Aristotle University of Thessaloniki, Thessaloniki, Greece; ^5^Laboratory of Physiology, School of Veterinary Medicine, Faculty of Health Sciences, Aristotle University of Thessaloniki, Thessaloniki, Greece; ^6^Academic Orthopaedic Department, Papageorgiou General Hospital and CORE Laboratory at CIRI-AUTh, AUTh Medical School, Thessaloniki, Greece

## Abstract

In this study, we aim to investigate the effective dose of botulinum neurotoxin A that results in paralysis of the sternocleidomastoid muscle for a minimum duration of 28 days in Wistar rats. This research is the first in a series of studies to investigate the value of botulinum toxin A in the healing of clavicle fractures through the temporary paralysis of the sternocleidomastoid. A surgical incision was made under general anaesthesia, and botulinum neurotoxin A in respective doses of 4 and 6 international units (IU) or normal saline in equivalent volumes were injected directly into the exposed muscle. Electromyography was conducted on days 0, 7, and 28 following substance administration to determine the extent of muscle paralysis. Electromyography on day 0 showed no paralysis in either group. Animals injected with neurotoxin all exhibited paralysis on days 7 and 28 that was weaker in the group injected with the smaller dose of 4 IU. One death occurred in the group injected with the higher dose (6 IU), whereas in the control group, no paralysis was seen. Botulinum neurotoxin A in a dose of 6 IU resulted in complete paralysis of the sternocleidomastoid in rats for a minimum of 28 days. A dose of 4 IU resulted in less potent paralysis but was safer in our research. Botulinum neurotoxin is a substance utilised in cosmetics and therapeutics for many years, yet research shows that its use can be expanded to target a wider range of pathologies. In this series of studies, we aim to explore the neurotoxin's applications on the treatment of clavicle fractures. To investigate this, we need to first establish the duration of its action on the sternocleidomastoid muscle.

## 1. Introduction

The sternocleidomastoid is one of the key muscles acting on the neck and head [1–3]. In humans, it originates from the manubrium of the sternum and the clavicle with two heads that merge into one belly to insert into the mastoid process and the superior nuchal line [1]. The muscle is responsible for head posture, rotation, inclination, and extension; moreover, its action on the sternum and the clavicles serve in inspiration, while it also assists the temporomandibular joint during mastication [1–3]. It is innervated by the cervical plexus and the accessory nerve; its blood supply derives from the occipital and superior thyroid arteries, and it is drained by the external jugular vein [1].

Pathologies of the muscle are related to several forms of torticollis and cervical dystonia, with treatment varying from conservative physiotherapy to surgical interventions [1, 4–6]. The use of botulinum toxin on the sternocleidomastoid to relieve or assist the treatment of torticollis has been proposed by several researchers and is an area attracting more and more interest [4–7].

Botulinum neurotoxins (BoNTs) are a family of exotoxins produced by *Clostridium* bacteria [8]. They are named neurotoxins because they act on cholinergic nerve terminals of the skeletal muscle and the autonomic nervous system to inhibit the release of the neurotransmitter acetylcholine resulting in paralysis [8]. This flaccid muscle paralysis is referred to as botulism, a hazardous manifestation with a potentially lethal outcome [8]. BoNTs are the toxins with the highest toxicity in existence; simultaneously, they are potent and neurospecific substances with limited diffusion at the injection site and with reversible action [8]. These features resulted into the commercialisation of BoNTs which have been increasingly used in medicine over the last 40 years [8]. Their paralysing effects on skeletal muscle are utilised to treat hypercontraction disorders such as blepharospasm and dystonias in the case of torticollis and spasticity [8, 9]. Moreover, their effect on the autonomic terminals allows for the expansion of their use for the treatment of hyperhidrosis or hypersalivation, urinary incontinence, migraine, and other pain syndromes; while a major use of BoNTs remains in the area of aesthetics with BoTox accounting for most BoNTs being injected annually [8–11]. Side effects are normally limited to injection site pain and irritation but can also involve manifestations as extreme as iatrogenic botulism [8, 12].

Botulinum neurotoxins are divided into subcategories (A–G) according to their individual characteristics, with BoNT A1 being the most effective but also the safest one and therefore the one utilised the most in medicine [8, 9, 12].

Duration of paralysis is variable and always type and dose-dependent [8]. The mode of administration and the type of nerve also play a role. Another parameter is the recipient organism, with humans being affected approximately three times longer than mice [8].

In the past, several studies have been conducted studying the effect of botulinum toxin on muscle paralysis. Normally, physicians use the toxin based on standardised guidelines but also based on their own clinical experience [8, 9, 12]. A full “mapping” of the toxin's duration on specific muscle groups for specific dosages is not yet available. In this study, we investigate the duration of paralysis of the sternocleidomastoid muscle in rats; specifically, we conducted a neurophysiological examination aiming to determine the safe dose of botulinum toxin that results in complete paralysis of the muscle for 28 minimum of days after intramuscular injection. To investigate this, 4 and 6 IU of botulinum toxin A were administered against the equivalent volumes of 0.9% normal saline solution (N.S.), and electromyography was be conducted on days 0, 7, and 28. The experimental doses of 4 and 6 IU were selected based on the already existing literature on quadriceps muscle paralysis in rats for 28 days [13]. Simultaneously, the experimental doses are nonlethal as they are within the reference ranges and were derived from the 2 IU/100 gr body mass rule [14–16]. Due to the growing use of botulinum neurotoxin A in cosmetics and therapeutics, determining the duration of the paralysing effect of specific doses on certain muscle groups can be useful in medicine and constitutes the significance of this study. The primary aim of this study, however, is to use the proposed dose of neurotoxin to achieve complete paralysis of the sternocleidomastoid muscle in rats with clavicle fractures and observe the effect on healing, i.e., whether the muscle paralysis allows for prompter and better fracture healing in the second study of this series.

## 2. Materials and Methods

The study was conducted on healthy, adult, male rats of the Albino Wistar breed, between 4 and 6 months of age, weighing between 350 and 450 grams. Rats were chosen since their anatomical configuration is similar to that of humans [17, 18]. Studies on the possible effect of gender on the effectiveness of botulinum toxin A muscle injection in rats are lucking. However, studies in humans revealed no difference in the dose and effectiveness of botulinum toxin A muscle injection between males and females [19]. To our knowledge, the majority of studies that examine muscle integrity and function after botulinum toxin A injection in rodents use male rats, which is why only male rats were included in the study [20–23].

The animals were divided into 4 groups, two groups of 3 and two groups of 2 animals based on the type of the injected substance, as well as its dose, and were marked accordingly for recognising purposes. The groups are given in Table 1.

During experimentation, the animals were bred and kept at the animal facility of the Experimental and Research Center of Papageorgiou G.H. Thessaloniki (accreditation number EL-54-BIOexp-01) and consumed food and water ad libitum. Procedures were in full accordance with the European Community Council directive 86/609/EEC. The experimentation received the approval of the Veterinary Directorate of Thessaloniki (approval number 346494 (1398)/27/07/2019) and followed the guidelines of the Aristotle University Ethics Committee for the use and care of laboratory animals.

**Figure 1:**
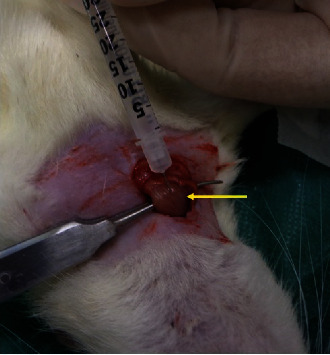
Exposure of right sternocleidomastoid muscle (yellow arrow) and direct intramuscular injection of substance.

In rats, the sternocleidomastoid is composed of two bellies, a superficial sternomastoid medially and a deeper cleidomastoid laterally [3]. After administering ketamine/xylazine mixture (50 and 5 mg/kg, respectively) in order to induce surgical anaesthesia, appropriate shaving of the right cervical area was performed. The right sternocleidomastoid muscle was located by palpation, and a surgical incision was made to expose it. All the procedures were performed by one surgeon under sterile conditions. A single bolus of botulinum toxin A or 0.9% NaCl was directly injected intramuscularly using a Hamilton syringe (Figure 1). The substance was injected in the middle portion of the muscle, which is the thickest part and the one in less proximity to the jugular vein's inferior bulb [19]. Botulinum toxin type A (BOTOX PD.INJ.SOL 100U/VIAL BTx1VIAL) was purchased from Allergan Pharmaceuticals Ltd. (FDA approved (24802.01.01). Three animals received 4 IU of botulinum toxin A, 3 animals received 6 IU, and 4 animals were injected with equivalent volumes of 0.9% normal saline.

**Figure 2:**
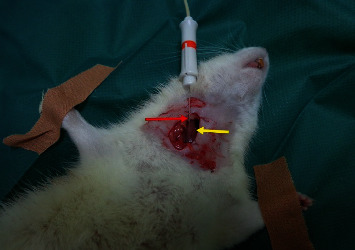
Recording electrode (red arrow) inserted into right cleidomastoid portion of sternocleidomastoid muscle (yellow arrow).

**Figure 3:**
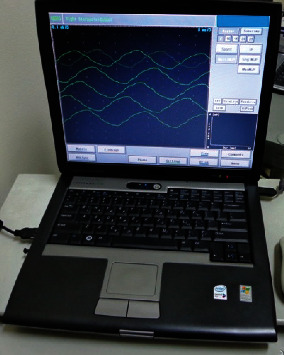
Electromyography equipment, Keypoint device.

Electromyography (EMG) was conducted under general anaesthesia on the 0, 7^th^, and 28^th^ day. The animals were positioned at left side recumbency; the right sternocleidomastoid muscle was exposed, and the needle (recording electrode) was inserted into the muscle's cleidomastoid portion (Figure 2). The electromyography equipment (Figure 3) used was a Keypoint device (Alpine Biomed Medical Devices, Skovlunde, Denmark v 5.11) with disposable concentric needle electrodes, size 37 mm × 26 G (Value Lie DCN, Natus Manufacturing Limited, IDA Business Park Gort, Co. Galway, Ireland). Following the electrophysiological control on day 28, the animals were euthanized by inhalation of CO_2_, a minimum-stress method [24, 25].

## 3. Results

All experimental animals showed normal appetite, behavior, and posture during the whole experimental period, and markers were used to monitor their physical condition. All animals were inspected daily for the presence of postoperative discomfort, stress, or pain, and no relevant findings occurred. One sudden death was noticed on the 3^rd^ postoperative day following 6 IU toxin administration.

Electromyography immediately after the toxin administration (day 0) revealed no spontaneous electrical activity (SEA) in any animal, something to be expected as the muscle does not have time to denervate within a few minutes/hours [26–29]. A week later, on day 7, the muscle showed signs of denervation depicted as continuous potentials in animals that received either 4 or 6 UI of toxin, a finding that coincides with literature [26–29].

**Figure 4:**
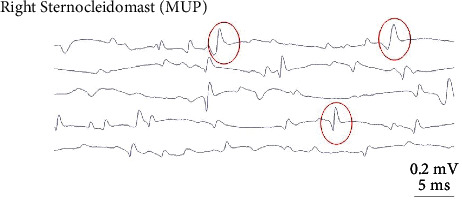
Electromyogram on day 28 postadministration of 4 IU botulinum toxin. Findings of SEA are circled in red.

**Figure 5:**
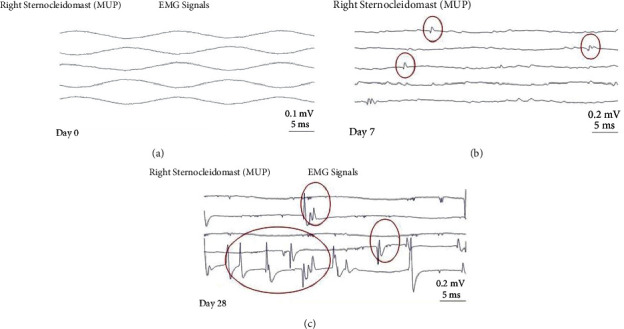
Electromyograms on days 0 (a), 7 (b), and 28 (c) after 6 IU botulinum toxin administration. Findings of SEA are circled in red.

**Figure 6:**
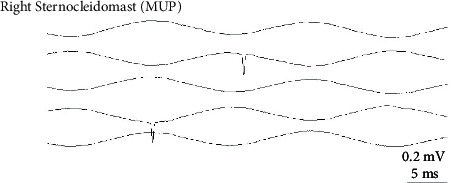
Electromyogram on day 28 after saline administration. No SEA finding.

Twenty-eight days postadministration, SEA was still present and was of similar intensity for all animals that received the 4 UI dose; whereas, it was more potent in animals that received the 6 IU dose (Figures 4 and 5). Animals that received normal saline showed no SEA as expected (Figure 6).

## 4. Discussion

The resting, healthy muscle is normally electrically silent with no spontaneous activity [26–28]. Spontaneous electrical activity is an indication of the chemical denervation of muscle fibres and a result of muscle paresis or paralysis. Denervation is a result of nerve damage/muscle paralysis and is graded from 1 to 4, 1 representing occasional signals and 4 showing signals filling the screen, corresponding to the degree of nerve damage/paralysis [26–28]. In the case of chemical denervation, SEA is usually expressed as fibrillation potentials (FP) or as positive sharp waves (PSW). Fibrillation potentials are triphasic waves with an initially positive deflection, a duration of 1–5 msecs and an amplitude of 10–200 *μ*V; they are released regularly with a frequency of 0.5–15 Hz and create a characteristic EMG sound that has been described as “rain on a tin roof.” Similarly, PSW represent biphasic waves with an initially sharp positive deflection and a subsequent prolonged negative deflection of an amplitude between 10 and 100 *μ*V, are regularly released with a frequency of 0.5–15 Hz, and produce a prolonged sound described as “claps of distant thunder” [26–28].

Spontaneous electrical activity is graded as follows:

--, no SEA
- +, presence of one potential, FP or PSW, for at least 400 msecs in a minimum of 2 EMG-controlled positions
- ++, 2 or more potentials, FP or PSW, recorded continuously and occupying 1/3 of the screen in 3 or more EMG-controlled positions
- +++, continuous potentials, FP or PSW, occupying half of the screen in all EMG-controlled positions
- ++++, continuous potentials, FP or PSW, completely occupying the screen in all EMG-controlled positions.

The duration and degree of muscle paralysis was studied in 10 adult male albino Wistar rats. Botulinum neurotoxin A1 was administered at the doses of 4 and 6 IU of equal volumes and was measured against 2 control groups injected with normal saline. Electromyography was conducted on the cleidomastoid muscle of the test subjects and the controls after the administration of botulinum toxin/N.S. at three different time points: 0, 7, and 28 days postinjection. The presence of pathologic spontaneous electrical activity (SEA) was studied. This paralysing effect appears on days 2-3 after the administration of the neurotoxin; the highest degree of paralysis is achieved in the 1^st^-2^nd^ week, depending on the recipient organism, the injection site, and the dose, and can be maintained for up to 4 months in skeletal muscle and for up to a year in autonomic nerve terminals [8]. Should this or similar research be extended to humans, the relevance of the measurements' timings lies within the fracture healing physiology. It is supported by literature that following the initial period after the fracture (days 0–5), the fibrocartillagenous callus starts to form between days 5 and 11, followed by the bony callus during days 11–28 [30]. As a result, measurements on days 0, 7, and 28 are selected to coincide with the timeline of bone healing.

Similar studies in the past measuring muscle paralysis after BoNT administration were conducted on the quadriceps muscle. This is innervated by the femoral nerve on which studies can be conducted with electrodes being injected safely into the nerve [24]. Contradictory to that, the sternocleidomastoid is innervated by CNXI, the spinal accessory nerve, as a result measurement of SEA which was in our case done on the muscle belly instead of the nerve which would be unsafe.

As expected, no animal showed SEA on day 0, as the effect did not have time to be exerted on the muscle. With respect to group A (4 IU), the muscle showed SEA of similar intensity on days 7 and 28; whereas, in group B (6 IU), the paralysing effects were of increased intensity on days 7 and 28. Nevertheless, it was in this same group that one death occurred on day 3. Although the cause could not be determined, it is speculated that it was related to the higher toxin dose as despite the most frequent peripheral paralysing effect of BoNTs, there have been reports of distant, central botulism due to retrograde (retroaxonal) transport or even afferent transport from ganglia to the central nervous system [8]. Although the toxin is not cytotoxic nor does it induce any axon/nerve degeneration, these long-distance effects that can be as central as in the ganglia, the spinal cord, and the brain stem can result in immediate death [8]. As a result, it seems that the 4 IU dose was safer and achieved paralysis for 28 days. It should be noted that in clinical practice, diffusion into the systemic circulation could depend on the speed of administration, the toxin's volume, its dose, and the injection site, as well as individual parameters such as gender (i.e., males have often thicker muscle), body mass index, and muscle anatomical factors [1, 8]. Finally, no paralysing effect was observed in the two control groups C and D that received NaCl.

The study has certain limitations, the most important of which is the small sample size. While aiming to use as little animals as reasonably achievable, we tested the effect of the toxin on 6 experimental and 4 control animals. The small size of our sample although in adherence to ethical standards does not allow for generalisation of our findings. A larger group would allow for collection of more data to be analysed. Another limitation of the study is cost; as this was not a funded project, the expenses for botulinum neurotoxin, the animals, and the renting of the laboratory as well as the equipment carried substantial costs. Finally, as explained above in more detail, due to the sternocleidomastoid's nervous supply originating from a cranial nerve, EMG investigations could not be done on the nerve itself, but recording electrodes were inserted into the muscle.

## 5. Conclusion

The potential value of BoNTs beyond the area of cosmetics is still under investigation. Although BoNTs have applications in certain muscle spasticity disorders, such as blepharospasm and dystonias (cervical torticollis), hyperhidrosis/hypersalivation, and in pain syndromes among others, accumulating evidence suggests that botulinum neurotoxin A1 has greater potential and knowledge of its effect on specific muscles can be useful in clinical practice. Further research should be carried out with respect to other body areas where temporary muscle paralysis could be purposeful aiming to utilise the toxin in a greater range of disorders. We will use this knowledge to investigate whether complete paralysis of the sternocleidomastoid muscle in Wistar rats injected with 4 IU aids the prompter healing of clavicle fractures.

## Figures and Tables

**Table 1:** Experimental and control groups.

Group	Animals	Substance injected
Group A	3 rats	4 IU botulinum toxin
Group B	3 rats	6 IU botulinum toxin
Group C	2 rats	0.9% NaCl
Group D	2 rats	0.9% NaCl

## Data Availability

The data used to support the findings of this study are available from the corresponding author upon request.
